# Synthesis of Nickel and Cobalt Ferrite-Doped Graphene as Efficient Catalysts for Improving the Hydrogen Storage Kinetics of Lithium Borohydride

**DOI:** 10.3390/ma16010427

**Published:** 2023-01-02

**Authors:** Petru Palade, Cezar Comanescu, Cristian Radu

**Affiliations:** 1 National Institute of Materials Physics, Atomistilor 405A, 077125 Magurele, Romania; 2 Faculty of Physics, University of Bucharest, Atomiștilor 405, 77125 Magurele, Romania

**Keywords:** hydrogen storage, lithium borohydride, catalyst, kinetics, graphene, energy, ferrite

## Abstract

Featuring a high hydrogen storage content of up to 20 wt%, complex metal borohydrides remain promising solid state hydrogen storage materials, with the real prospect of reversible behavior for a zero–emission economy. However, the thermodynamic barriers and sluggish kinetics are still barriers to overcome. In this context, nanoconfinement has provided a reliable method to improve the behavior of hydrogen storage materials. The present work describes the thermodynamic and kinetic enhancements of LiBH_4_ nanoconfined in MFe_2_O_4_ (M=Co, Ni) ferrite-catalyzed graphene host. Composites of LiBH_4_-catalysts were prepared by melt infiltration and investigated by X-ray diffraction, TEM, STEM-EDS and TPD. The role of ferrite additives, metal precursor treatment (Ar, Ar/H_2_) and the effect on hydrogen storage parameters are discussed. The thermodynamic parameters for the most promising composite LiBH_4_-graphene-NiFe_2_O_4_ (Ar) were investigated by Kissinger plot method, revealing an E_A_ = 127 kJ/mol, significantly lower than that of neat LiBH_4_ (170 kJ/mol). The reversible H_2_ content of LiBH_4_-graphene-NiFe_2_O_4_ (Ar) after 5 a/d cycles was ~6.14 wt%, in line with DOE’s target of 5.5 wt% storage capacity, while exhibiting the lowest desorption temperature peak of 349 °C. The composites with catalysts treated in Ar have lower desorption temperature due to better catalyst dispersion than using H_2_/Ar.

## 1. Introduction

The safe and effective production, storage and transport of hydrogen energy remains a current challenge. Among potential hydrogen sources, solid-state hydrogen storage materials such as simple and complex metal hydrides have been extensively investigated over the past few decades [1]. A series of shortcomings plague their wide use in mainstream energy systems, such as sluggish kinetics and high thermodynamic barriers, which translate into a high practical dehydrogenation temperature. Possible means to mitigate these drawbacks refer to nanosizing, nanoconfinement, and usage of additives and catalysts, among others [2].

When nanoconfined in the pores of a proper scaffold, the behavior of borohydrides and alanates features marked improvements over their pristine counterparts [1,2]. On one hand, the particle agglomeration and growing during cycling are inhibited, which is particularly important since higher temperatures are required during a/d measurements. This strategy can guarantee a better kinetic behavior of the investigated composites, which showed increased dehydrogenation rates. On the other hand, the nanoporous scaffold facilitates gas diffusion during cycling, contributing to the overall enhanced behavior.

A key step in engineering competent nanocomposites for energy storage is to ensure the active hydrogen storage material is confined uniformly at the nanoscale, so that there are no outer-host processes like crystal growth which would adversely affect the hydrogen storage performance. The majority of previously-reported works deal with confinement of complex hydrides in previously-prepared hosts, which impairs some degree of inhomogeneity. Many nanoporous substrates were used as scaffolds: 2D-structured silica, carbon, carbon nanobowls, mesoporous carbon, and graphene [3]. Graphene nanosheets are 2D materials that have been extensively used in energy storage applications, both in pristine and catalyzed form. Among most utilized catalysts are metal fluorides, chlorides oxides Fe_3_O_4_ [4] etc.

Among investigated species nanoconfined in porous scaffolds are LiBH_4_ [5,6,7,8,9,10,11,12,13,14,15], Ca(BH_4_)_2_ [16], or LiAlH_4_ [17]. There are, however, some scarce reports where nanosized complex hydride was synthesized and showed unexpectedly high storage capacity of 12% in case of LiBH_4_ for instance, without the additional support of a porous scaffold [18]. Various enhancements can be obtained in low temperature synthesis by solid-gas reaction [19], extensive study of pressure or temperature dependence of decomposition [20] and extensive reversibility studies [21]. These in-extenso studies also revealed some of the plausible intermediates occurring during dehydrogenation, such as Li_2_B_12_H_12_ and possibly other crystallographic forms of LiBH_4_ as well [22]. Other aspects with high practical importance such as the oxidation of borohydride groups occurring upon exposure to oxygen–containing atmosphere or functional groups grafted onto the nanoporous support have also been described [23]. Modern investigation techniques like QENS (neutron vibrational spectroscopy and quasielastic neutron scattering) have been used to probe the dynamic properties of borohydride anion, with relevance to hydrogen storage measurements [24]. Other mixed oxides have shown good catalytic activity for ammonia borane methanolysis [25,26]. Other potential strategies to improve complex hydride behavior have been reviewed recently [27], while CoFe_2_O_4_ and NiFe_2_O_4_ have been investigated as catalysts for complex hydrides, especially for complex hydrides like lithium borohydride LiBH_4_ [28] and alkali metal alanates LiAlH_4_ [29,30] and NaAlH_4_ [31].

However, oftentimes the activity of nanosized catalysts dispersed in porous scaffolds yields results which are hard to predict and moreover, even differ between various research groups. A possible reason is that, even though the chemical nature of the catalyst may be similar, its dispersion within the scaffold is not homogeneous and even the chemical composition of the catalyst can be altered during HEBM (high energy ball milling). In the current work, we address the inhomogeneity issue of the catalyst dispersion inside graphene 2D sheets by an in-situ catalyst preparation (NiFe_2_O_4_, CoFe_2_O_4_) starting from a stoichiometric mixture of corresponding metal nitrates. The thermal decomposition of metal precursors was carried out under two different conditions: in Ar flow or H_2_/Ar flow. A comparison of catalyst morphology revealed that the Ar flow treatment leads to a better catalyst dispersion inside graphene nanosheets, hence producing a more potent catalyst. Additionally, the in-situ production of MFe_2_O_4_ (M=Fe, Co) stems from a bottom-up approach that would ensure homogeneous catalyst dispersion and a more controllable kinetic behavior of confined hydride species. The different gas flow approaches (inert–Ar or reducing H_2_/Ar) allowed further evaluation of metal ferrites (CoFe_2_O_4_, NiFe_2_O_4_–under Ar) and intermetallics/alloys (FeNi, Fe, CoFe_2_, Fe_3_C, Co_3_C–obtained under reducing atmosphere H_2_/Ar). In all investigated cases, the ferrite catalysts showed enhanced kinetic behavior of the LiBH_4_@catalyst nanocomposites, and superior to the intermetallic catalysts obtained under reducing conditions, providing a useful starting point for further investigation of in-situ generated ferrite catalysts for hydrogen production.

## 2. Materials and Methods

Starting materials for samples preparations were: lithium borohydride, LiBH_4_ (>95%, Sigma Aldrich, St. Louis, MO, USA), graphene nanoplatelets (surface area 500 m^2^/g, powder size <2 μm, thickness of few nm, Sigma Aldrich), iron (III) nitrate nonahydrate, Fe(NO_3_)_3_⋅9H_2_O, (>99.9%, Sigma Aldrich), nickel (II) nitrate hexahydrate Ni(NO_3_)_2_⋅6H_2_O (>98.5%, Sigma Aldrich), cobalt (II) nitrate hexahydrate Co(NO_3_)_2_⋅6H_2_O (>98%, Sigma Aldrich), absolute ethanol (99.9%, Sigma Aldrich). The catalysts based on cobalt ferrite and nickel ferrite supported on graphene were prepared and used as supports for subsequent LiBH_4_ melt impregnation. Stoichiometric amounts of Fe(NO_3_)_3_⋅9H_2_O and Ni(NO_3_)_2_⋅6H_2_O or Co(NO_3_)_2_⋅6H_2_O corresponding to the final loading of graphene with either 15 wt% of NiFe_2_O_4_ or CoFe_2_O_4_ have been impregnated by incipient wetness method (in 5–7 impregnation steps) from absolute ethanol solutions of corresponding nitrates. The concentrations of the solutions were 0.045 M ethanol solutions of Ni(NO_3_)_2_.6H_2_O or Co(NO_3_)_2_.6H_2_O, and 0.09 M for Fe(NO_3_)_3_.9H_2_O, and were used in stoichiometric amount to yield MFe_2_O_4_ (M=Ni, Co). The impregnated samples were subjected to thermal treatments either in Argon (Ar) or 5% Hydrogen in Argon (further noted in the text as: H_2_Ar) gas flow (treatment temperature 620 °C, treatment time 4 h, gas flow rate 100 mL/min). The CoFe_2_O_4_/graphene and NiFe_2_O_4_/graphene catalysts synthesized in Ar or H_2_Ar gas flow were mixed with LiBH_4_ in the mass ratio 1:1 by prolonged time grinding under dry inert atmosphere using pestle and mortar. Afterwards they were hydrogenated at 300 °C in hydrogen gas with 99.9999% purity under 80 atm H_2_ pressure. This temperature was above the melting point of LiBH_4_ (270 °C) allowing an intimate mixing between lithium borohydride and the supported catalysts. The samples processing was carried out in MBraun LabStar (Garching bei Munchen, Germany) glove box under purified re-circulated Argon (<1 ppm O_2_, <1 ppm H_2_O) during all stages of sample manipulation. X-Ray diffraction investigation was performed using D-8 Advance Bruker diffractometer (Bruker, Karlsruhe, Germany) with Cu K-alpha radiation. During X-ray diffraction measurements the samples were covered with polymeric foil in order to avoid oxidation. TEM images were obtained with JEM-2100 analytical transmission electron microscope (JEOL, Tokyo, Japan) operated at 200 kV endowed with dispersive X-ray spectrometer. For TEM measurements, the samples were dispersed in hexane using high power device (VCX 750 Sonics, Newtown, CT, USA) and afterwards were deposited on grids by drying the hexane. A commercially available Sievert volumetric apparatus (Advanced Material Corporation, AMC Pittsburg, Pittsburg, KS, USA) was used for hydrogen absorption and desorption measurements at a particular temperature or with a temperature ramp rate (thermal programmed desorption TPD).

## 3. Results

### 3.1. X-ray Diffraction Analysis

Five composite samples were prepared and investigated in the present work: (i) LiBH_4_-graphene, (ii) LiBH_4_-(NiFe_2_O_4_/graphene catalyst heat treated in Ar flow), (iii) LiBH_4_-(NiFe_2_O_4_/graphene catalyst heat treated in H_2_Ar flow), (iv) LiBH_4_-(CoFe_2_O_4_/graphene catalyst heat treated in Ar flow) (v) LiBH_4_-(CoFe_2_O_4_/graphene catalyst heat treated in H_2_Ar flow). The catalysts contain 15 wt% NiFe_2_O_4_ or CoFe_2_O_4_ dispersed on graphene.

**Figure 1:**
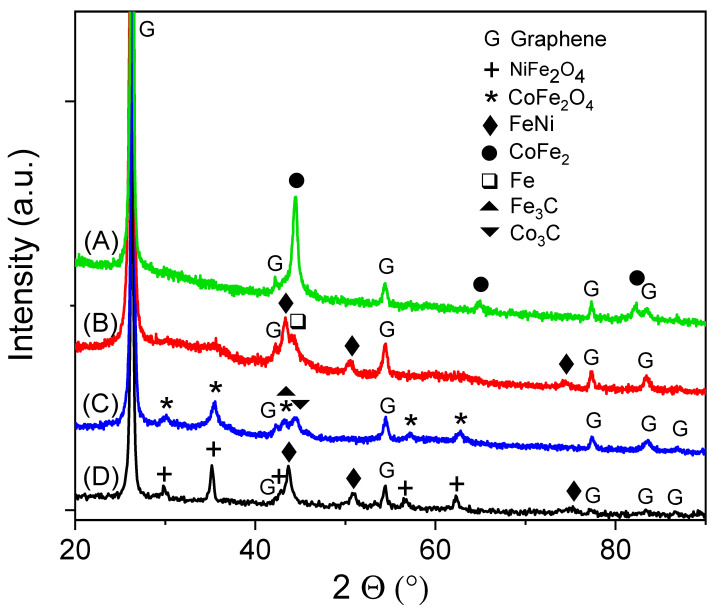
X-ray diffraction data for synthesized catalysts supported on graphene: (A) 15 wt% CoFe_2_O_4_/graphene (treated in H_2_Ar flow), (B) 15 wt% NiFe_2_O_4_/graphene (treated in H_2_Ar flow), (C) 15 wt% CoFe_2_O_4_/graphene (treated in Ar flow), (D) 15 wt% NiFe_2_O_4_/graphene (treated in Ar flow).

For simplicity, in the following notations were used: (A) 15 wt% CoFe_2_O_4_/graphene (treated in H_2_Ar flow) as CFO-G-H_2_Ar, (B) 15 wt% NiFe_2_O_4_/graphene (treated in H_2_Ar flow) as NFO-G-H_2_Ar, (C) 15 wt% CoFe_2_O_4_/graphene (treated in Ar flow) as CFO-G-Ar, and (D) 15 wt% NiFe_2_O_4_/graphene (treated in Ar flow) as NFO-G-Ar. The XRD patterns of the catalysts synthesized in the present work are depicted in Figure 1. In all XRD diagrams the dominant contribution belongs to graphene, the peak from 2Θ: 26° being much higher than any of the other peaks. Besides graphene, sample NFO-G-Ar contains NiFe_2_O_4_ (ICDD file 04-005-6361) and FeNi (ICDD file 04-021-6318). The formation of FeNi is supported by the reducing effect of the carbon matrix. By contrast, the sample CFO-G-Ar contains mainly CoFe_2_O_4_ (ICDD file 00-066-0244), but also small amounts of carbide phases Fe_3_C (ICDD file 04-008-9572) and Co_3_C (ICDD file 04-003-4355). Regarding the samples treated under reducing flux (H_2_Ar), the ferrite phase MFe_2_O_4_ (M=Co, Ni) is no longer visible in XRD diffractogram, and full reduction to metallic alloy/intermetallic MFe_x_ (x = 1,2) can be observed. The sample NFO-G-H_2_Ar contains only FeNi and Fe-bcc, besides the main contribution from graphene. As previously mentioned in the literature, the hydrogenation of NiFe_2_O_4_ leads to the formation of FeNi and Fe and this observation holds true for reduction of nanoconfined nickel ferrite in the particular case when using a graphene support matrix. In the case of CoFe_2_O_4_ the treatment in H_2_ or H_2_Ar (hydrogen + argon) mixture generates CoFe_2_ (ICDD file 04-016-4643). Hence, the sample CFO-G-H_2_Ar contains only CoFe_2_ besides the main graphene contribution. The XRD peaks corresponding to NiFe_2_O_4_, CoFe_2_O_4_, FeNi and Fe are relatively wide, suggesting grains in the nanometric range. For clarity, a summary of phase identification of these catalyzed graphene supports can be found in the Table S1 (Supplementary Materials).

**Figure 2:**
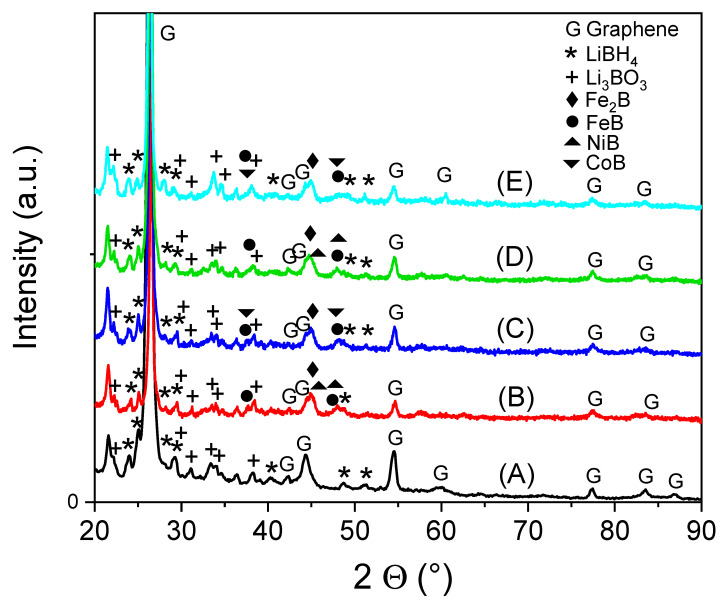
X-ray diffraction data for synthesized mixtures of LiBH_4_ with catalysts supported on graphene after re-hydrogenation: (A) LiBH_4_-graphene, (B) LiBH_4_-NFO-G-Ar, (C) LiBH_4_-CFO-G-Ar, (D) LiBH_4_-NFO-G-H_2_Ar, (E) LiBH_4_-CFO-G-H_2_Ar.

Figure 2 represents the X-ray diffractograms of LiBH_4_ mixed with CoFe_2_O_4_ and NiFe_2_O_4_ catalysts supported on graphene. The samples were extracted after five cycles of hydrogen absorption/desorption being in final re-hydrogenated (absorbed) state. During measurements the samples were covered with polymeric foil in order to avoid oxidation. The peaks from from 2Θ of about 22° and 36° belong to this foil. Expectedly, the dominant contribution from the XRD pattern belongs to graphene. Additionally, in the sample LiBH_4_-G were evidenced not only the peaks of LiBH_4_ (ICDD file 01-084-8599) but also an important contribution from Li_3_BO_3_ (ICDD file 00-018-0718) as effect of surface oxidation. After hydrogen absorbtion/desorption cycles, LiBH_4_ interacts with the ferrites or intermetallics contained in the samples, forming borides. FeB (ICDD file 04-004-2772) and Fe_2_B (ICDD file 04-002-9005) appeared in all samples which contain supported catalysts. The samples LiBH_4_-CFO-Ar and LiBH_4_-CFO-H_2_Ar contain also CoB (ICDD file 04-004-2683) whereas samples LiBH_4_-NFO-Ar and LiBH_4_-NFO-H_2_Ar present also NiB (ICDD file 01-074-1207), besides iron borides, LiBH_4_ and Li_3_BO_3_. The borides peak are broad, clearly indicating very small grains. A summary of the phase identification of used catalyst (after 5 a/d cycles) is presented for clarity also in Table S1 (Supplementary Materials).

### 3.2. Morphological and Compositional Investigation by TEM and STEM-EDS

**Figure 3:**
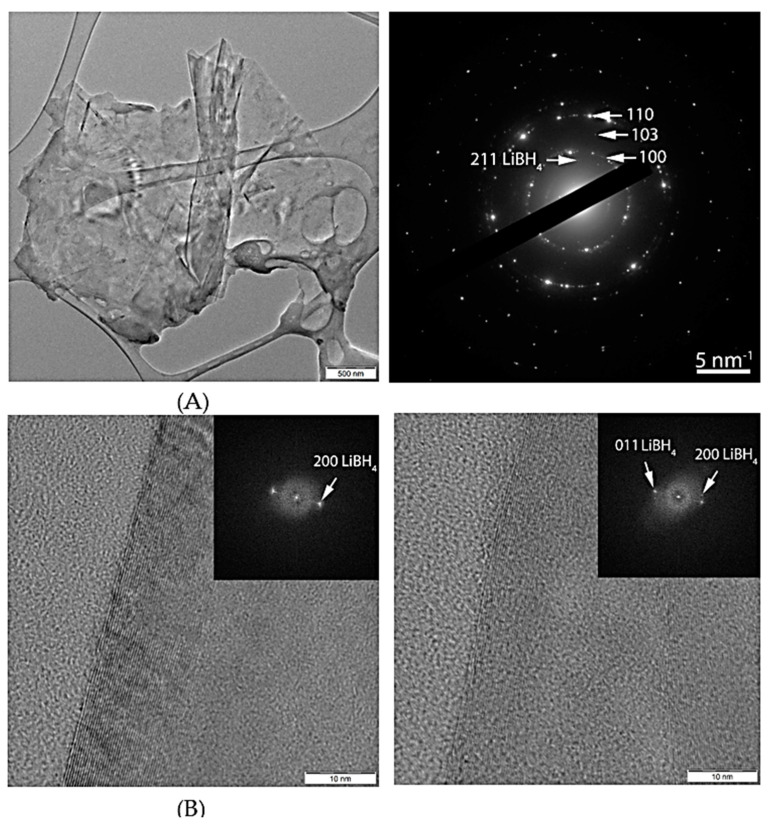
Low magnification (**A**) and higher magnification (**B**) TEM images for LiBH_4_-G sample along with SAED pattern (**A**). The corresponding FFT pattern is in inset (**B**).

TEM images for the samples LiBH_4_-G, LiBH_4_-NFO-G-Ar, LiBH_4_-NFO-G-H_2_Ar, LiBH_4_-CFO-G-Ar depict large sheets of graphene, with lateral dimensions of a few microns. The selected area diffraction patterns (SAED) of all 4 samples reveal the presence of graphene (rings corresponding to graphite interplanar distances were identified). Also the spots corresponding to LiBH_4_ were detected. TEM images at higher magnification revealed elongated crystalline zones with 7–14 nm thickness (Figure 3). Measurements on the FFT of these images concluded that the nanocrystals consist of LiBH_4_.

**Figure 4:**
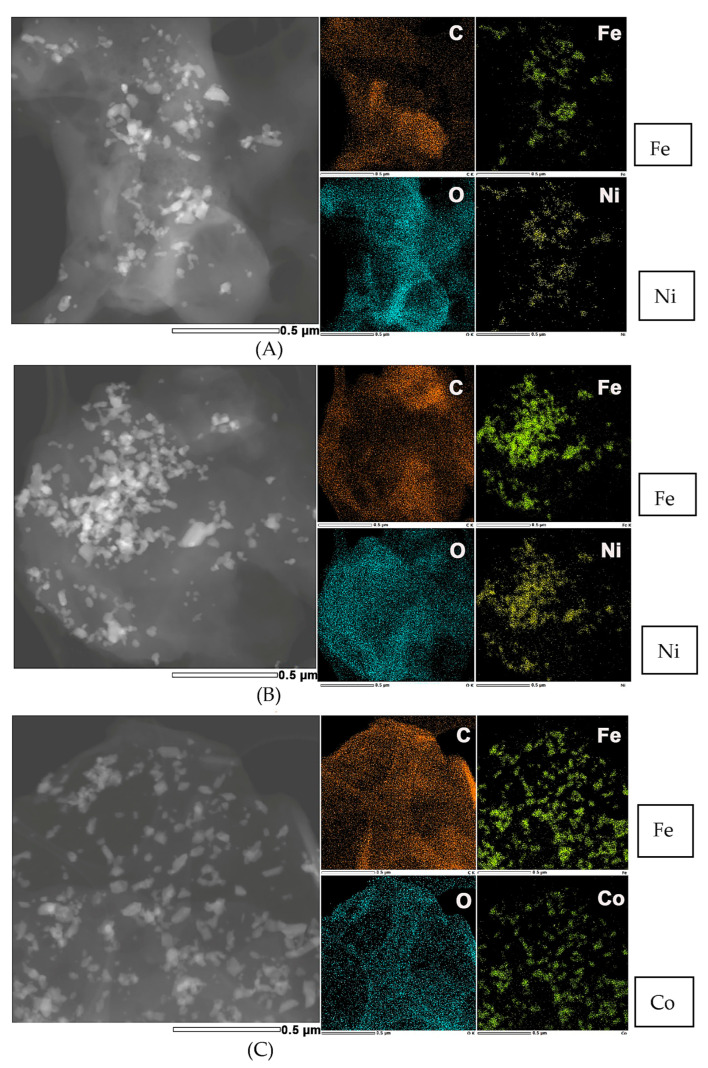
STEM-EDS measurements for samples LiBH_4_-NFO-G-Ar (**A**), LiBH_4_-NFO-G-H_2_Ar (**B**), and LiBH_4_-CFO-G-Ar (**C**). The scale bar on all individual figures is 0.5 µm.

STEM-EDS measurements for samples LiBH_4_-NFO-G-Ar, LiBH_4_-NFO-G-H_2_Ar, LiBH_4_-CFO-G-Ar, and LiBH_4_-CFO-G-H_2_Ar (Figure 4) proved the presence of nanoparticles containing metals besides LiBH_4_ and graphite. In all samples the nanoparticles have sizes of the order of tens of nanometers (in a wide range 20–80 nm) and have irregular shapes. In samples LiBH_4_-NFO-G-Ar and LiBH_4_-CFO-G-Ar the nanoparticles are well separated, although they seem to be more uniformly distributed in LiBH_4_-CFO-G-Ar. The sample LiBH_4_-NFO-G-H_2_Ar contains nanoparticles more likely to form large clusters with high particles densities, while in other areas these clusters are absent. Their composition was confirmed by the EDS mapping to be: Fe-Ni (for LiBH_4_-NFO-G-Ar, and LiBH_4_-NFO-G-H_2_Ar), and Fe-Co (for LiBH_4_-CFO-G-Ar), with slightly higher iron concentration.

### 3.3. Hydrogen Storage Property Measurements

**Figure 5:**
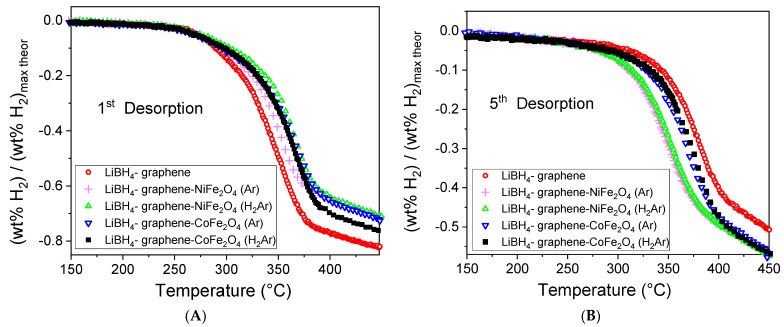
Thermal programmed desorption (TPD) measurements with temperature ramp rate (2 °C/min) for the first hydrogen release (**A**) and after 5 cycles of hydrogen desorption/absorption (**B**).

Investigation of hydrogen desorption behavior using temperature ramp rate of 2 °C/min (thermal programmed desorption—TPD) was performed using AMC Sievert volumetric apparatus (Advanced Material Corporation, AMC Pittsburg, Pittsburg, KS, USA). The samples were manipulated in inert and dry atmosphere in glove box and transferred in the sample holder of AMC apparatus using the same facility. The samples were first degassed in vacuum at about 100 °C, but temperatures above 150 °C have been avoided during degassing due to the risk of hydrogen desorption before the envisaged release measurement for as–prepared samples. Hydrogen desorption measurements with temperature ramp rate (TPD) were performed both for first desorption (Figure 5A) (when the fresh sample was just loaded in the device) and after five cycles of hydrogenation in order to evaluate the behavior and composition of investigated nanocomposites (Figure 5B).

The theoretical maximum value of hydrogen amount released during LiBH_4_ decomposition into LiH and B is 13.8 wt% H_2_ [1,2]. In Figure 5 the experimental wt% H_2_ was normalized to this value. LiBH_4_ without graphene or catalyst addition released a normalized value of only 0.25 for desorption measurement with 2 °C/min up to 450 °C. For subsequent desorption, the sample was loaded with H_2_ after each hydrogen desorption at 450 °C and 80 atm H_2_ for prolonged time (24 h) in order to ensure that the sample reached the maximum level of absorbed hydrogen. One can observe that even though the first LiBH_4_-G decomposition is th e fastest among all samples, the same sample behaves worst at the saturated behavior after 5 a/d cycles. This confirmed the catalytic role of NiFe_2_O_4_ and CoFe_2_O_4_ addition to graphene, which improves the hydrogen desorption process. Hydrogen desorption with temperature ramp rate of 2 °C/min up to 450 °C is not enough to release all the hydrogen from samples. In order to achieve this goal, the desorption was allowed longer time at the final temperature of 450 °C. The corresponding desorption kinetics curves were provided in electronic Supplementary Materials for the first desorption (Figure S1) and for the fifth desorption (Figure S2).

## 4. Discussion

**Figure 6:**
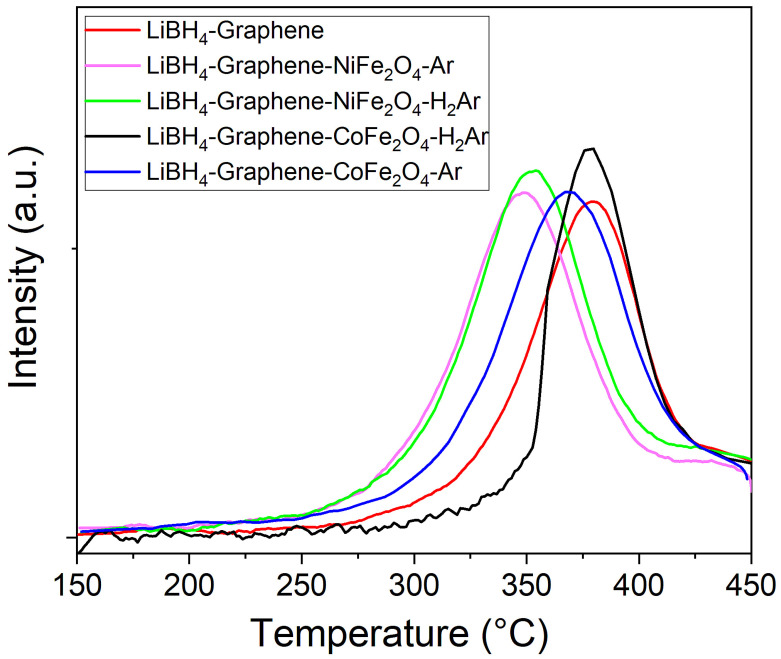
Desorption peak temperature for samples: LiBH_4_-graphene, LiBH_4_-NFO-G-Ar, LiBH_4_-CFO-G-Ar, LiBH_4_-NFO-G-H_2_Ar, LiBH_4_-CFO-G-H_2_Ar.

Using the TPD from Figure 5B one can derive the desorption peak temperature for all samples after 5 a/d cycles, as shown in Figure 6.

As depicted in Figure 6, after 5 a/d cycles, the maximum desorption peak temperature belongs to LiBH_4_-graphene sample that proved the advantages of doping graphene with cobalt or nickel ferrite in order to improve hydrogen desorption kinetics of LiBH_4_. In Table 1 are gathered the values of normalized hydrogen desorption amount for 1st and 5th de-hydrogenation along with desorption peak temperature for the latter. The normalized released amount values correspond both to the partial desorption (up to 450 °C with 2 °C/min temperature ramp rate when desorption is not completed due to kinetic limitations) and after extensive time, enough to complete desorption at the final temperature of 450 °C. Desorption peak temperature is lower for LiBH_4_-NFO sample compared with LiBH_4_-CFO sample for catalysts treated both in Ar and H_2_Ar flow.

As one may observe, the graphene supported catalysts based on nickel ferrite proved better than that based on cobalt ferrite for improving hydrogen desorption kinetics of LiBH_4_. On the other hand, LiBH_4_ mixed with catalysts heat treated in Ar flow have lower desorption peak temperature than their counterparts annealed in H_2_Ar flow both for cobalt and nickel ferrite supported on graphene. One should recall from STEM-EDS (Figure 4) that the catalysts treated in H_2_Ar preferentially formed large metal clusters whereas the ones treated in Ar were better dispersed. In Table 1 one can observe larger values obtained for the normalized hydrogen amount released in the 1st de-hydrogenation compared with 5th de-hydrogenation for all samples. This behavior is understandable due to formation of FeB, Fe_2_B, NiB and CoB, as shown in the X-ray diffractograms, diminishing the amount of LiBH_4_ from the samples. However, there is a clear advantage of the boride presence, which is due to their catalytic effect. Interestingly, even for the complete desorption in the first cycle, LiBH_4_-G desorbed only 0.95 normalized amount whereas all the catalyzed samples reached the maximum theoretical allowed amount. For the 5th de-hydrogenation, the normalized amount is lower for LiBH_4_-G compared with that corresponding to catalyzed samples both for TPD up to 450 °C with 2 °C/min temperature ramp rate, and for complete desorption. This behavior proves once again the efficiency of using cobalt and nickel ferrite to produce catalyst-supported graphene scaffolds, which can effectively improve hydrogen desorption of nanoconfined LiBH_4_.

**Figure 7:**
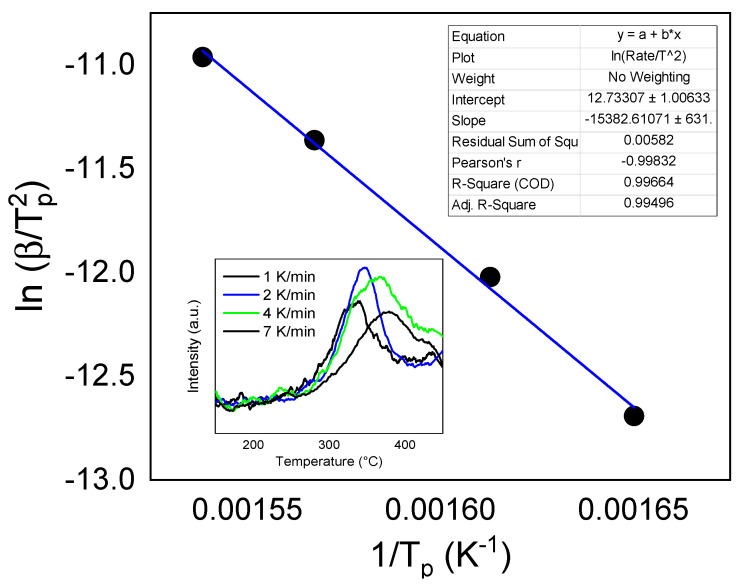
Kissinger plot of LiBH_4_-G-NFO-Ar sample, where the inset corresponds to TPD curves measured at different heating rates.

The desorption temperature peak for LiBH_4_-NFO-G-Ar (lithium borohydride mixed with nickel ferrite supported on graphene heat treated in Ar flow) is the lowest among all samples. For this sample, TPD measurements were performed with different temperature ramp rate of 1 K/min, 2 K/min, 4 K/min and 7 K/min up to 450 °C (see inset of Figure 7). The corresponding desorption temperature peaks were used to obtain the Kissinger plot, which allowed evaluation of the hydrogen desorption activation energy from the slope of the linear fit (Figure 7). The obtained value of 127 kJ/mol for the activation energy is significantly lower than that of bulk LiBH_4_, reported in the range 146 kJ/mol [15]–170 kJ/mol [4], which again confirms the catalytic role of nanosized metal ferrites synthesized.

## 5. Conclusions

The present work investigated the improved efficiency of LiBH_4_ desorption kinetics brought about by new synthesized catalysts based on Ni- and Co-ferrites supported on graphene scaffolds. These catalysts were prepared from graphene and the constituent nitrates by performing subsequent heat treatment in argon or (hydrogen + argon) flow at 620 °C. The composites LiBH_4_-catalysts were prepared by prolonged mixing under protective atmosphere followed by melt infiltration under hydrogen pressure. Dehydrogenation measurements confirmed that both Ni- and Co-ferrite catalyzed graphene nanocomposites show faster desorption kinetics than pristine graphene. LiBH_4_-(NiFe_2_O_4_/graphene) using nickel ferrite catalyst synthesized in Ar flow has the lowest desorption temperature peak of 349 °C, whereas the same composition, but using treatment in H_2_/Ar, presents the highest reversible storing capacity of 6.14 wt% H_2_. The Kissinger plot for LiBH_4_-(NiFe_2_O_4_/graphene/Ar) revealed an activation energy of 127 kJ/mol. The kinetic improvement can be traced to the Fe, Ni and Co borides formed during hydrogenation. The composites with catalysts treated in Ar have lower desorption temperature due to better catalyst dispersion than using H_2_/Ar, when catalyst clustering was observed by STEM-EDS measurements. The Ni–ferrite NiFe_2_O_4_ was more effective than Co–ferrite CoFe_2_O_4_ in lowering the decomposition temperature of catalyzed LiBH_4_ composites, and also more effective than metal borides and carbides identified in the reaction mixture as potential catalysts. Additionally, the current work highlights the role of metal ferrites, metal boride and carbide phases acting as hydrogenation catalysts, providing useful information on the actual state of catalytic sites during cycling. Considering the promising results exhibited by graphene-supported MFe_2_O_4_ (M=Co, Ni), the ferrite–based supports will be further investigated in catalytic de–/rehydrogenation studies of other complex hydrides and RHCs.

## Figures and Tables

**Table 1:** Normalized hydrogen desorption amount up to 450 °C with 2 °C/min temperature ramp and for long desorption time at final temperature of 450 °C for first desorption and after 5 cycles of hydrogenation together with the desorption peak temperature for the 5th dehydrogenation.

Sample	Normalized 1st Des. <450 °C/Complete	Normalized 5th Des. <450 °C/Complete	T Des. Peak (°C)
LiBH_4_-G	0.83/0.95	0.51/0.86	379.5
LiBH_4_-G-NFO-Ar	0.72/0.99	0.57/0.89	349
LiBH_4_-G-NFO-H_2_Ar	0.71/0.99	0.58/0.91	353
LiBH_4_-G-CFO-Ar	0.72/0.99	0.56/0.87	369
LiBH_4_-G-CFO-H_2_Ar	0.77/0.99	0.57/0.87	378

## Data Availability

The raw data presented in the current paper can be obtained from the corresponding author upon a reasonable formal request.

## Supplementary Materials

The following supporting information can be downloaded at: https://www.mdpi.com/article/10.3390/ma16010427/s1, Hydrogenation studies in Sievert-type apparatus (Figures S1 and S2), Electron microscopy and EDAX (Figures S3–S6), Powder diffraction data (XRD) (Figures S7–S15) and Summary of support and LiBH4@support phases as identified by powder XRD (Table S1).
