# Supplementary material for: Synthesis of Nickel and Cobalt Ferrite-Doped Graphene as Efficient Catalysts for Improving the Hydrogen Storage Kinetics of Lithium Borohydride
Source: Materials (Basel). 2023 Jan 2;16(1):427. doi: 10.3390/ma16010427 (PMC9822379; doi:10.3390/ma16010427)
Supplement: Supplementary file 1 [file materials-16-00427-s001.zip › materials-2114630-supplementary.pdf]

Synthesis of Nickel and Cobalt Ferrite-Doped Graphene as Efficient Catalysts for Improving the Hydrogen Storage Kinetics of Lithium Borohydride – Supplementary Information

Electronic Supporting information for:

Synthesis of Nickel and Cobalt Ferrite-Doped Graphene as Efficient Catalysts for Improving the Hydrogen Storage Kinetics of Lithium Borohydride

Petru Palade [1,†], Cezar Comanescu [1,2,*,†] and Cristian Radu [1]

[1] National Institute of Materials Physics, Atomistilor 405A, 077125 Magurele, Romania

[2] Faculty of Physics, University of Bucharest, Atomiștilor 405, 77125 Magurele, Romania

* Correspondence: cezar.comanescu@infim.ro

1. **Hydrogenation studies in Sievert-type apparatus**
   1.1. **Figure S1.** First desorption for all synthesized samples……2
   1.2. **Figure S2.** The 5th desorption for all synthesized samples……3
2. **Electron microscopy and EDAX**
   2.1. **Figure S3.** TEM, and elemental concentration for $LiBH_4$–Graphene–$NiFe_2O_4$–Ar……4
   2.2. **Figure S4.** EDAX data for $LiBH_4$–Graphene–$NiFe_2O_4$–Ar (after re-hydrogenation)……4
   2.3. **Figure S5.** TEM, and elemental concentration for $LiBH_4$–Graphene–$CoFe_2O_4$–Ar……5
   2.4. **Figure S6.** EDAX data for $LiBH_4$–Graphene–$CoFe_2O_4$–Ar (after re-hydrogenation)……5
3. **Powder diffraction data (XRD)**
   3.1. **Figure S7**. XRD diffractogram for **Graphene–$NiFe_2O_4$–Ar** catalyzed support……6
   3.2. **Figure S8**. XRD diffractogram for **Graphene–$NiFe_2O_4$–H_2Ar** catalyzed support……6
   3.3. **Figure S9**. XRD diffractogram for **Graphene–$CoFe_2O_4$–Ar** catalyzed support……7
   3.4. **Figure S10**. XRD diffractogram for **Graphene–$CoFe_2O_4$–H_2Ar** catalyzed support……7
   3.5. **Figure S11**. XRD diffractogram for **$LiBH_4$–Graphene**……8
   3.6. **Figure S12**. XRD diffractogram for **$LiBH_4$–Graphene–$NiFe_2O_4$–Ar–ABS** nanocomposite……9
   3.7. **Figure S13**. XRD diffractogram for **$LiBH_4$–Graphene–$NiFe_2O_4$–H_2Ar–ABS** nanocomposite……9
   3.8. **Figure S14**. XRD diffractogram for **$LiBH_4$–Graphene–$CoFe_2O_4$–Ar–ABS** nanocomposite……10
   3.9. **Figure S15**. XRD diffractogram for **$LiBH_4$–Graphene–$CoFe_2O_4$–H_2Ar–ABS** nanocomposite….10
4. **Summary of support and $LiBH_4$@support phases as identified by powder XRD**
   4.1. **Table S1**. Phase composition of catalyzed supports (*as-prepared*), and after 5 a/d cycles (*used catalysts*)……11

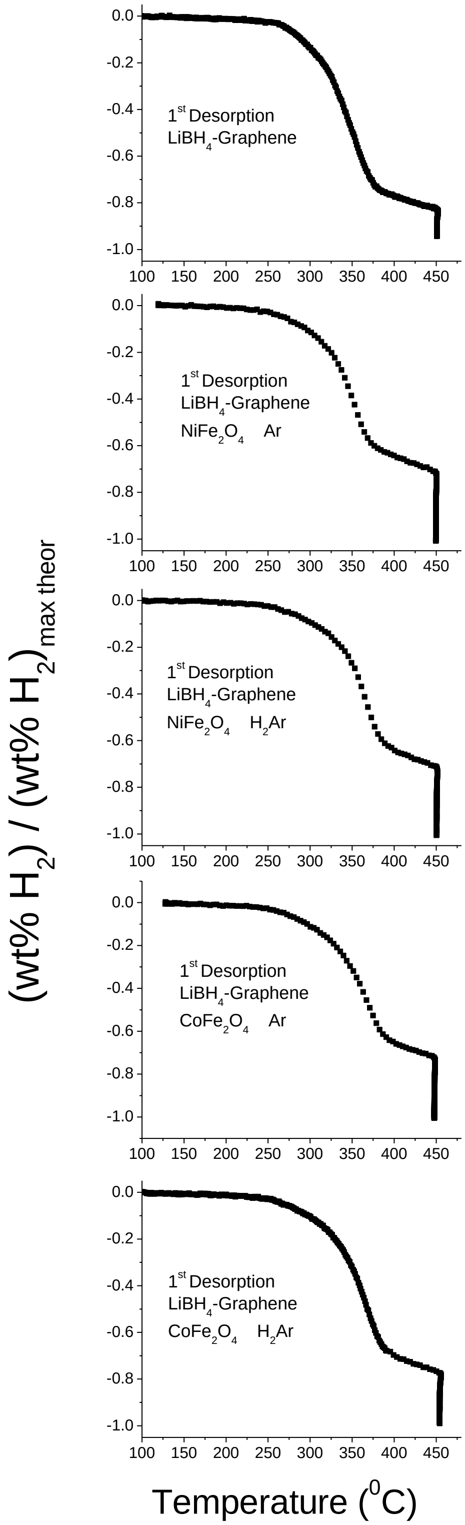

Figure S1. First desorption for all synthesized samples (top–to-bottom): $LiBH_4$–Graphene; $LiBH_4$–Graphene–$NiFe_2O_4$–Ar; $LiBH_4$–Graphene–$NiFe_2O_4$–H_2Ar; $LiBH_4$–Graphene–$CoFe_2O_4$–Ar and $LiBH_4$–Graphene–$CoFe_2O_4$–H_2Ar.

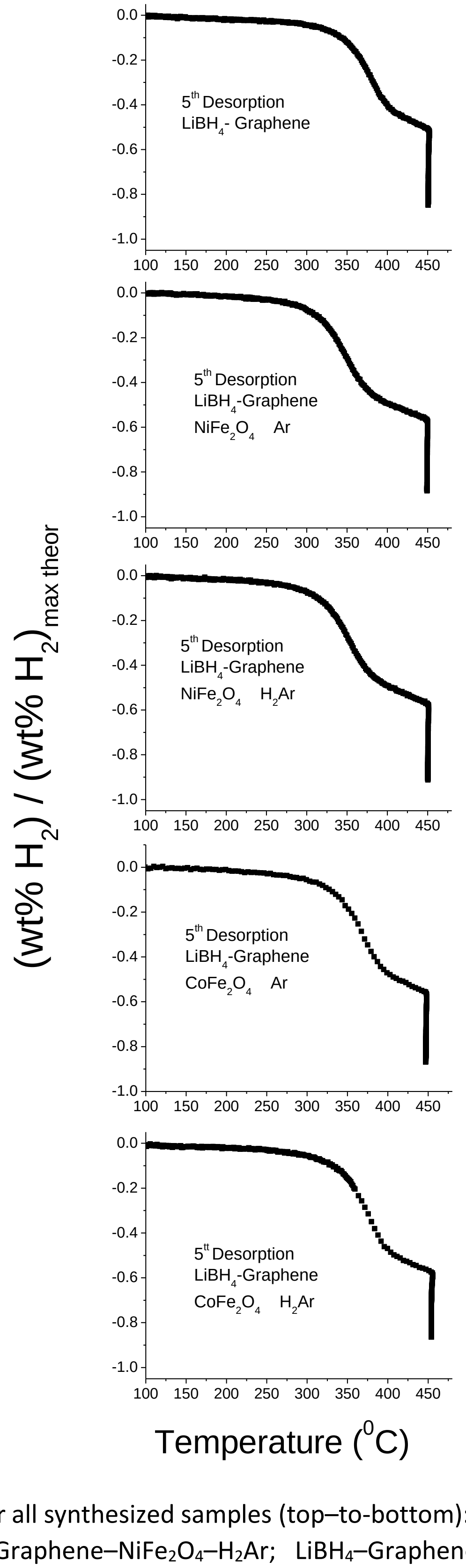

Figure S2. The 5th desorption for all synthesized samples (top–to-bottom): $LiBH_4$–Graphene; $LiBH_4$–Graphene–$NiFe_2O_4$–Ar; $LiBH_4$–Graphene–$NiFe_2O_4$–H_2Ar; $LiBH_4$–Graphene–$CoFe_2O_4$–Ar and $LiBH_4$–Graphene–$CoFe_2O_4$–H_2Ar.

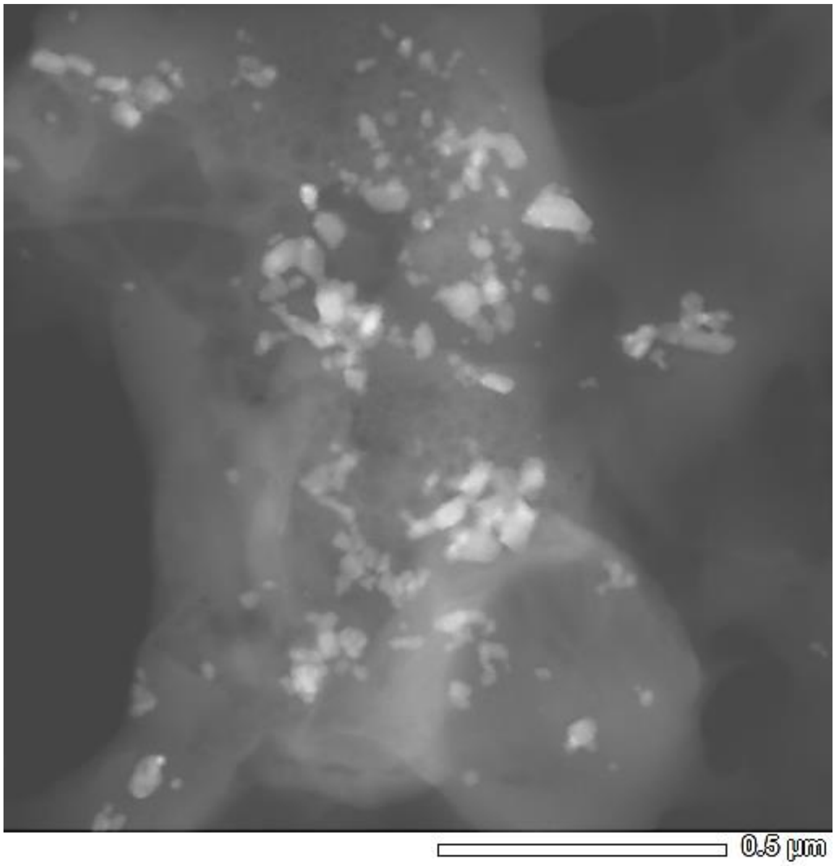

Element	At. composition (%)
Fe	0.71
Ni	0.34

Ratio Fe/Ni: 2.08

Figure S3. TEM, and elemental concentration for $LiBH_4$–Graphene–$NiFe_2O_4$–Ar (after re-hydrogenation).

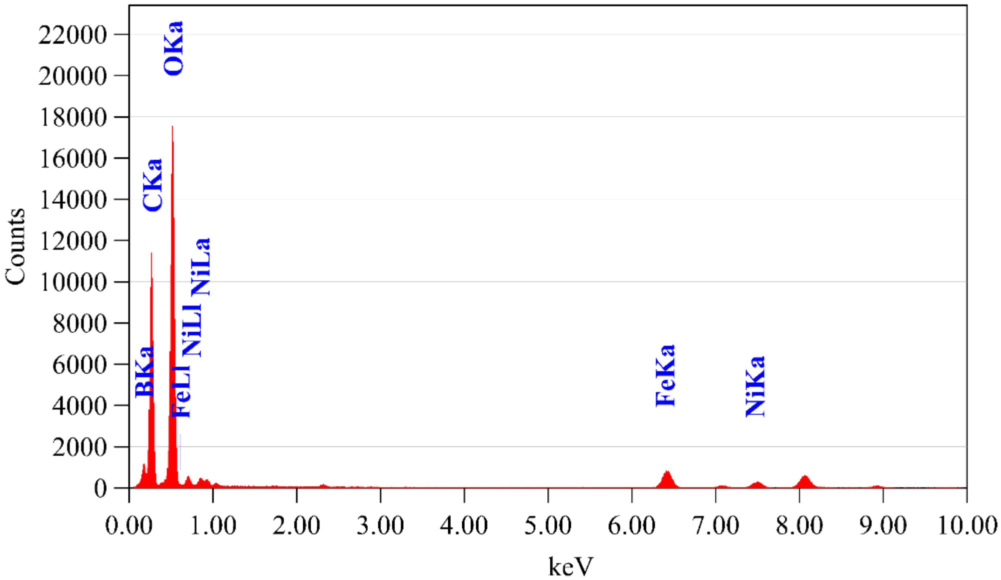

Figure S4. EDAX data for $LiBH_4$–Graphene–$NiFe_2O_4$–Ar (after re-hydrogenation).

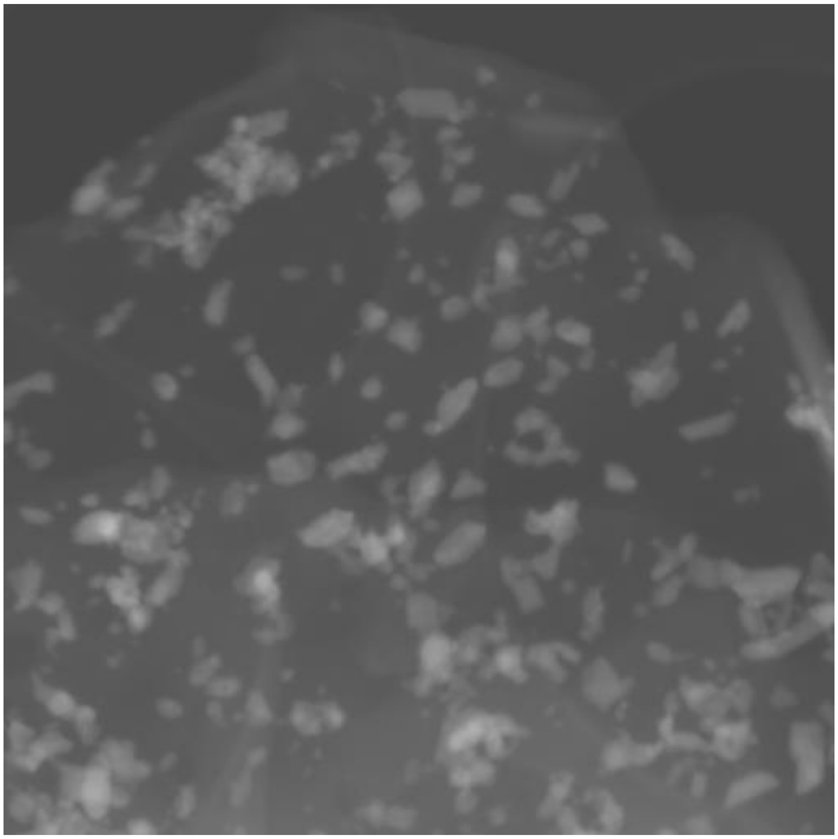

Element	At. composition (%)
Fe	1.55
Co	0.73

Ratio Fe/Co: 2.12

Figure S5. TEM, and elemental concentration for $LiBH_4$–Graphene–$CoFe_2O_4$–Ar (after re-hydrogenation).

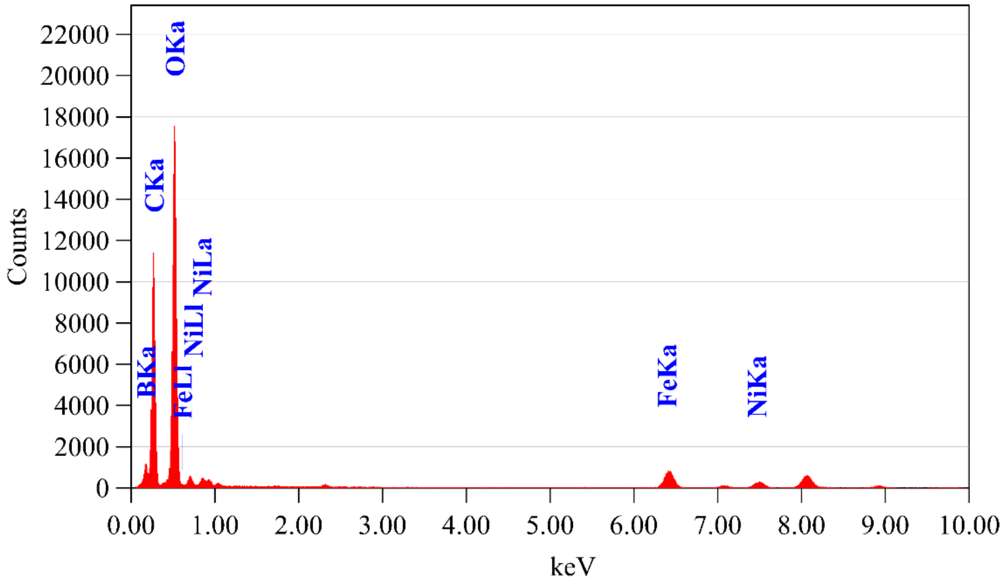

Figure S6. EDAX data for $LiBH_4$–Graphene–$CoFe_2O_4$–Ar (after re-hydrogenation).

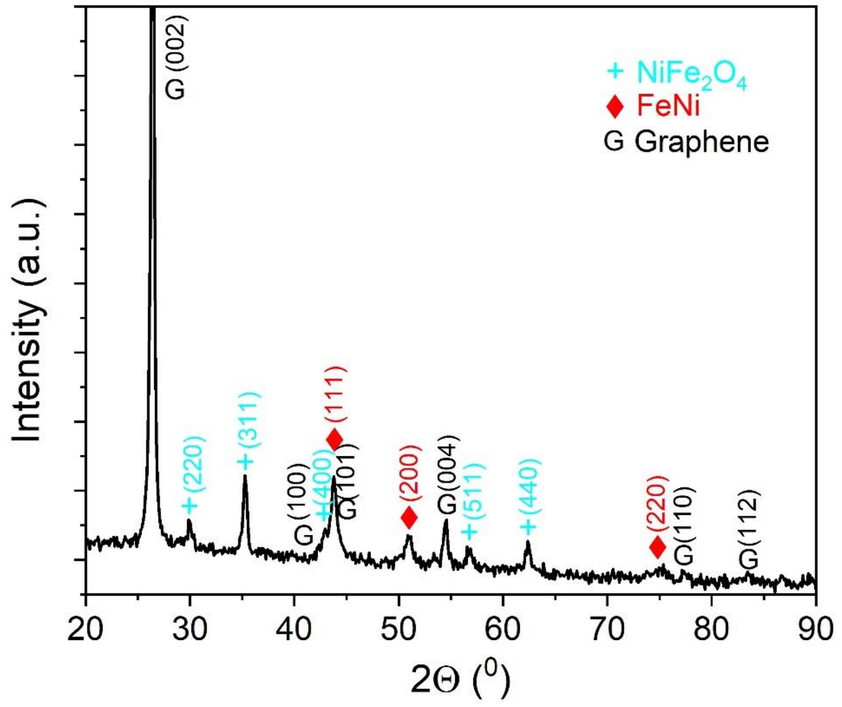

Figure S7. XRD diffractogram for **Graphene–$NiFe_2O_4$–Ar** catalyzed support.

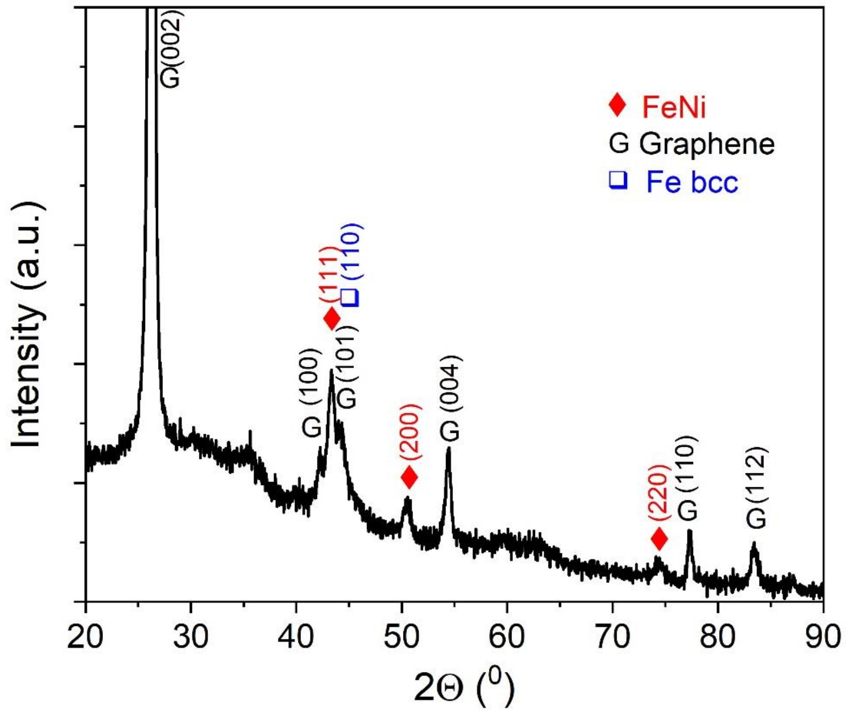

Figure S8. XRD diffractogram for **Graphene–$NiFe_2O_4$–H_2Ar** catalyzed support.

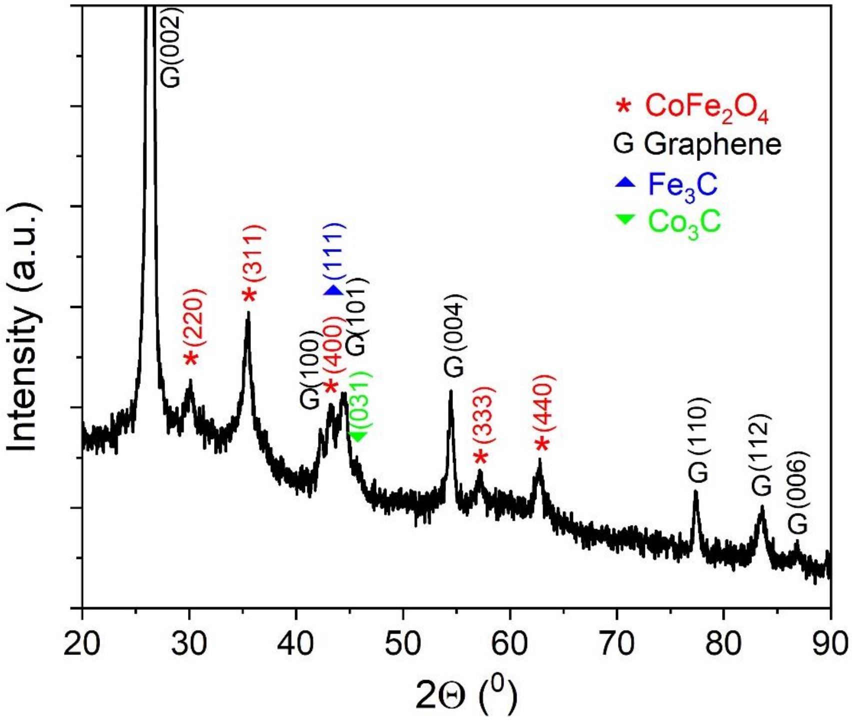

Figure S9. XRD diffractogram for **Graphene–$CoFe_2O_4$–Ar** catalyzed support.

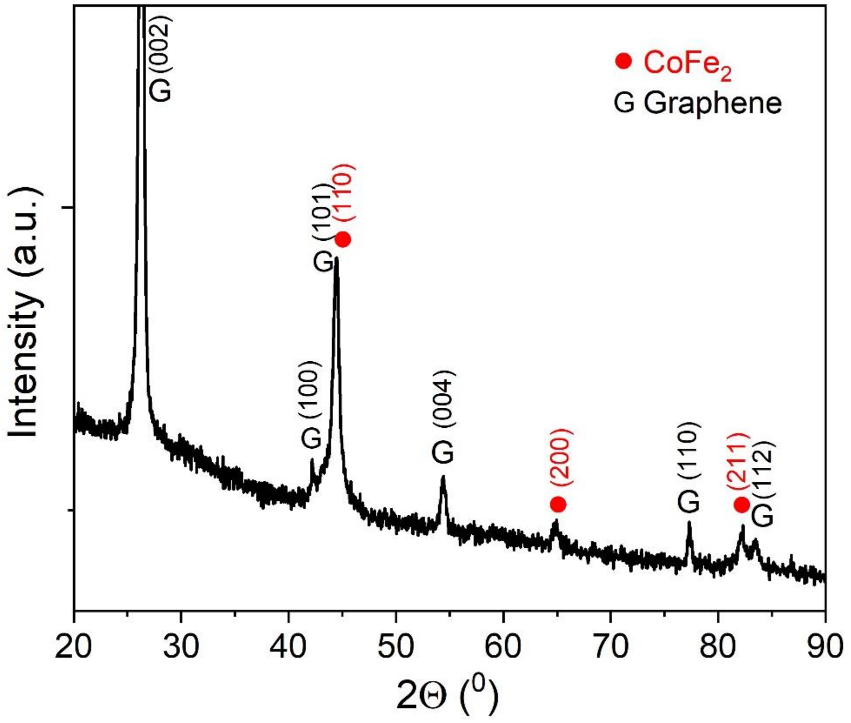

Figure S10. XRD diffractogram for **Graphene–$CoFe_2O_4$–H_2Ar** catalyzed support.

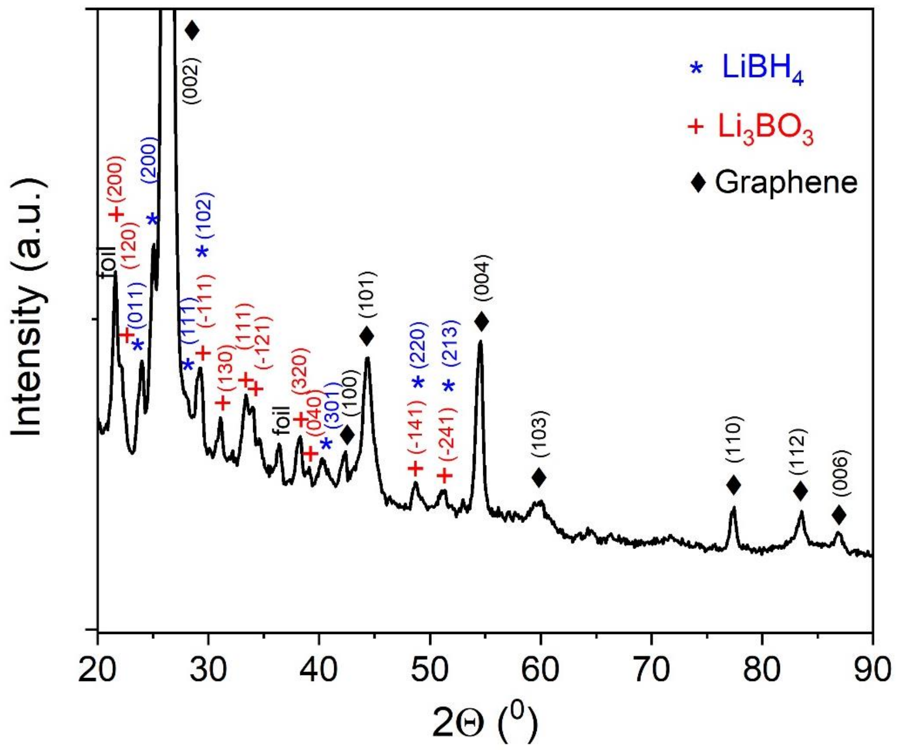

Figure S11. XRD diffractogram for **$LiBH_4$–Graphene**.

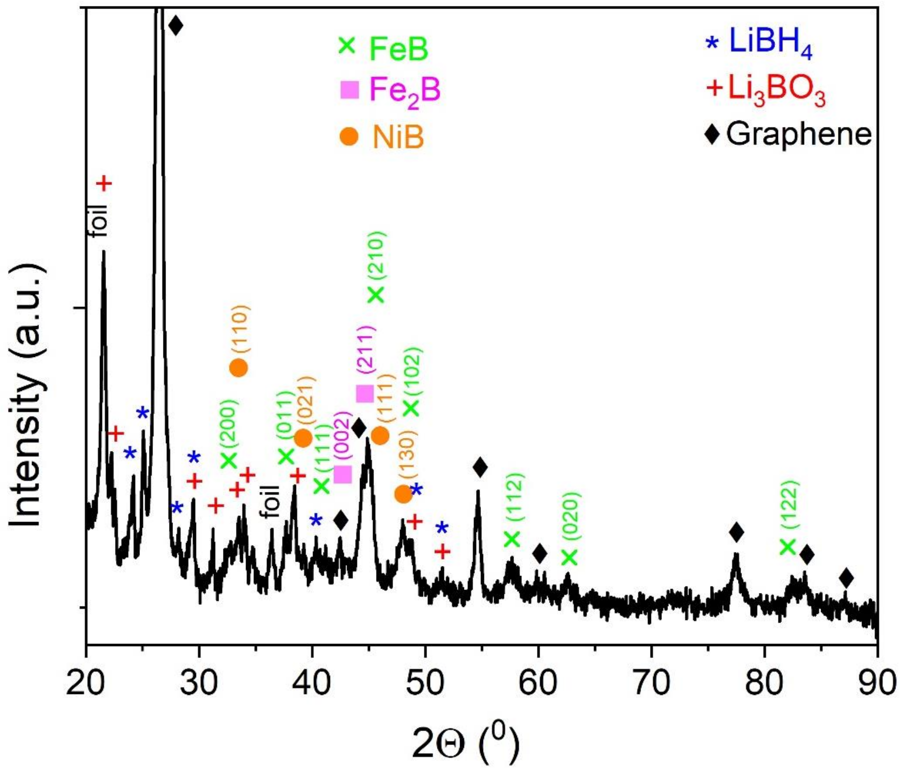

Figure S12. XRD diffractogram for **$LiBH_4$–Graphene–$NiFe_2O_4$–Ar–ABS** nanocomposite.

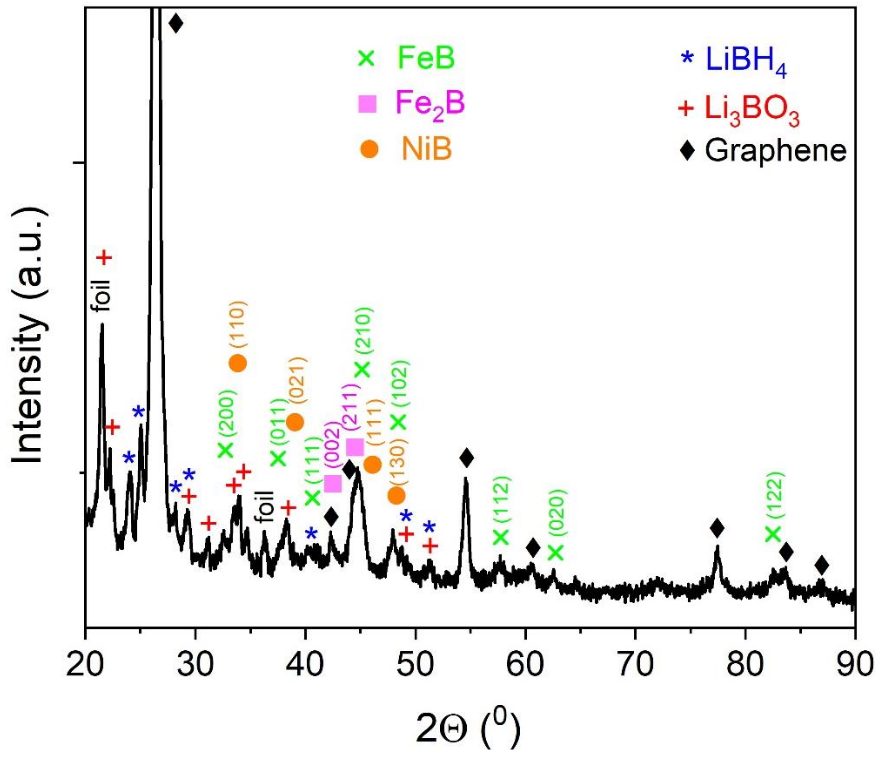

Figure S13. XRD diffractogram for **$LiBH_4$–Graphene–$NiFe_2O_4$–H_2Ar–ABS** nanocomposite.

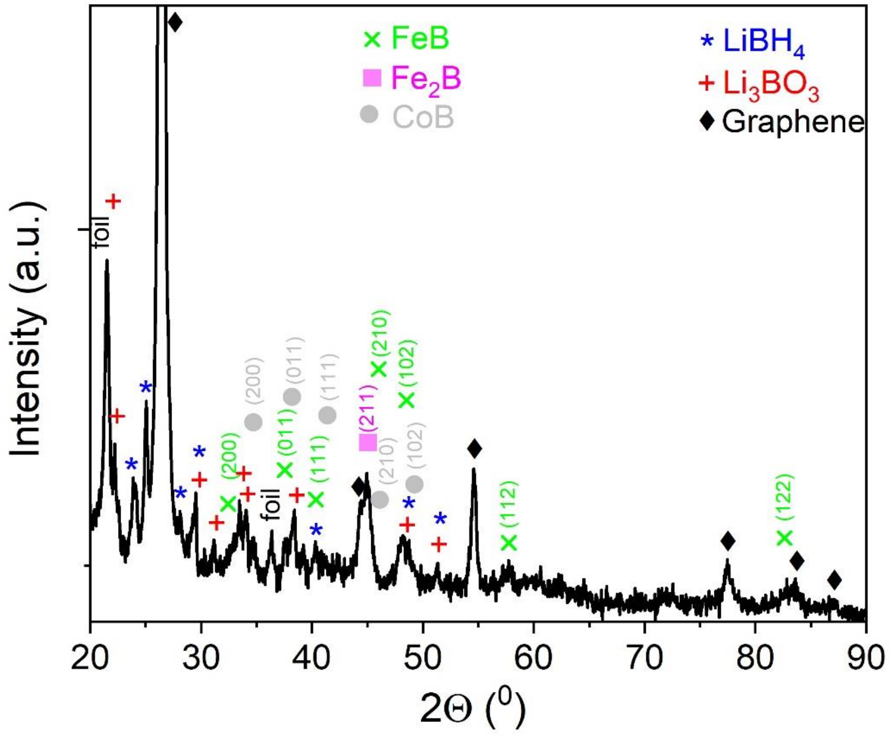

Figure S14. XRD diffractogram for **$LiBH_4$–Graphene–$CoFe_2O_4$–Ar–ABS** nanocomposite.

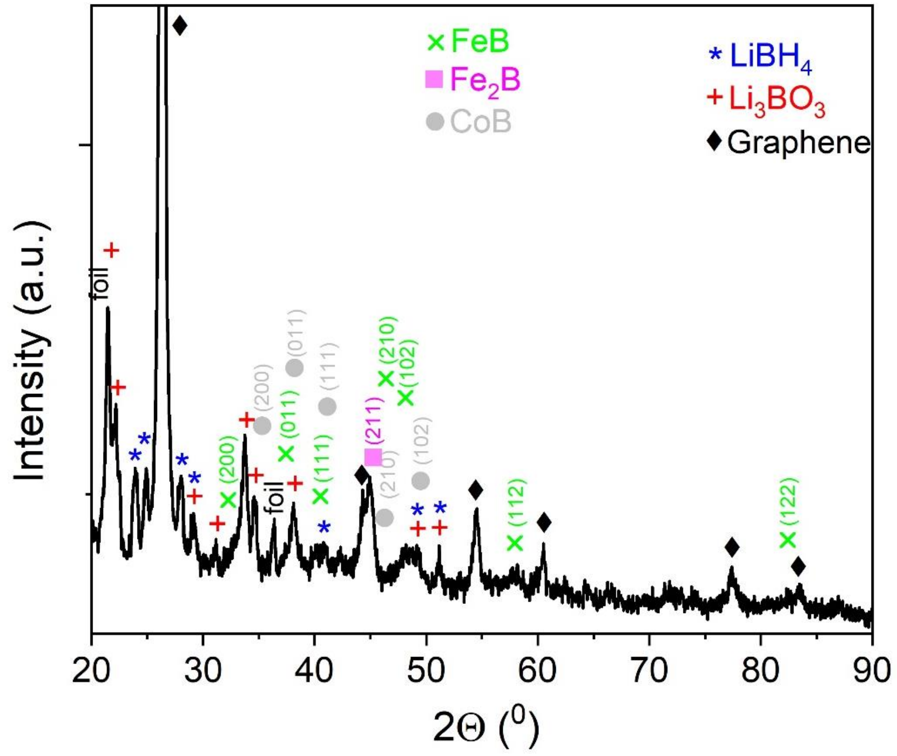

Figure S15. XRD diffractogram for **$LiBH_4$–Graphene–$CoFe_2O_4$–H_2Ar–ABS** nanocomposite.

Table S1. Phase composition of catalyzed supports (as-prepared), and after 5 a/d cycles (used catalysts), as deduced from XRD spectra presented in Figures S7–S15. The graphene support (G) is present in all investigated samples.

**Support** type	Phases in catalyzed support (XRD)	Phases in **$LiBH_4$@catalyzed support–ABS** rehydrogenated, after 5 a/d cycles
**Graphene–$NiFe_2O_4$–Ar**	(G), $NiFe_2O_4$, FeNi	(G), $LiBH_4$, Li_3BO_3, FeB, Fe_2B, NiB
**Graphene–$NiFe_2O_4$–H_2Ar**	(G), FeNi, Fe-bcc	(G), $LiBH_4$, Li_3BO_3, FeB, Fe_2B, NiB
**Graphene–$CoFe_2O_4$–Ar**	(G), $CoFe_2O_4$, Fe_3C, Co_3C	(G), $LiBH_4$, Li_3BO_3, FeB, Fe_2B, CoB
**Graphene–$CoFe_2O_4$–H_2Ar**	(G), $CoFe_2$	(G), $LiBH_4$, Li_3BO_3, FeB, Fe_2B, CoB
